# Metagenomics analysis of mice gut microbiome to unravel the role of metal exposure and piperine

**DOI:** 10.1099/acmi.0.000653.v3

**Published:** 2024-02-20

**Authors:** Ravidarshdeep Kaur, Dhirendra Pratap Singh, Rakesh Rawal

**Affiliations:** ^1^​ Department of Biochemistry and Forensic Science, University School of Sciences, Gujarat University, Ahmedabad-380009, Gujarat, India; ^2^​ Division of Biosciences, ICMR- National Institute of Occupational Health, Meghani Nagar, Ahmedabad-380016, Gujarat, India

**Keywords:** arsenic, metagenomics, metal toxicity, piperine

## Abstract

The gut and intestinal microbiota consists of trillions of microorganisms inhabiting the human gastrointestinal tract. It plays a crucial role in human health leading to understanding the dynamic crosstalk of host-microbe interaction in the gut and has become necessary for the detection, prevention, or therapy of diseases. Gut microbiota deviations are linked with many diseases, suggesting that various pathways involved in immunity, energy, lipid, and glucose metabolism are affected. Further, it is also altered by external insults such as metal toxicity, antibiotics and pesticides. Heavy metals like arsenic, mercury, cadmium and chromium are some of the well-studied classes of environmental pollutants. Mouse models have become the model of choice for most studies in this emerging field, as they allow perturbations in the gut microbiota to be studied in a controlled experimental setup. Here, we investigate the composition and diversity of intestinal microbes utilizing cecal samples from different intervention groups: arsenic exposure (As(III)), arsenic and piperine co-administration (As +Pp), piperine per se and control group. We obtained DNA samples from these groups and performed PCR amplification and sequencing of the 16S V3-V4 region. The findings showed shift in microbial composition and abundance among different intervention groups, revealing taxa that may contribute to the microbial diversity.

## Data Summary

The data analysed has been represented in the manuscript and raw data has been submitted to NCBI under the BioProject PRJNA1060264.

## Introduction

Metagenomics analysis has revolutionized our understanding of microbial communities by providing comprehensive insights into their genetic composition and functional potential. This powerful approach involves the direct sequencing and analysis of DNA extracted from environmental samples, enabling researchers to explore the collective genomes of microorganisms without the need for microbial cultivation [1]. Knowing that more than 99 % of prokaryotes in the environment cannot be cultured in the laboratory, the metagenomics approach is the culture-independent analysis of a mixture of microbial genomes based on sequencing [2]. Formerly just thought of as a digestive organ, the gastrointestinal tract is now being studied for its potential influence on human health and disease. The Human Gut Microbiome Project has increased our understanding of the bacterial community that lives within the human intestine [3].

The gut microbiota plays an active role in metabolism of nutrients, enhancement of the immune system and inhibition of potential pathogens. Studies on the gut bacterial community composition shows essential relationship between gut microbiota and good health [4]. Alterations in the gut microbiota of control group as compared to treated ones, may have important implications for the health of the animals, and thus factors that modify the gut microbiota like environmental contaminants such as heavy metal toxicity are of great interest and arsenic is important among them [5, 6]. Multiple studies have investigated the effect of arsenic exposure and shift in gastrointestinal microbiota diversity [7, 8]. Heavy metals and other contaminants like arsenic, cadmium, lead, and mercury have become a serious health hazard in recent years. They have been connected to harmful outcomes such oxidative stress, carcinogenesis, DNA damage, and immune system weakening. Numerous recent research have suggested that exposure to heavy metals may be linked to gut microbial dysbiosis [9]. However, fewer studies have investigated the restoration of the diversified microbial community due to induced arsenic toxicity in mouse models. Here, we have profiled the intestinal microbiome of Swiss albino mice perturbed with arsenic and investigated the potential role of piperine. It is a potential alkaloid that may help in the alteration of gut bacterial communities and dysbiosis [10]. Here, its administration is characterized to identify significant changes in the microbial communities of species and to provide a target for the therapy against heavy metal toxicity.

## Methods

### Animal experimentation

A total of 20 Swiss albino, male mice of between 8–12 weeks with initial weight 22–24 g were procured form Zydus Healthcare Changodar, Ahmedabad, Gujarat after receiving the institution’s ethical approval. CPCSEA guidelines were followed throughout the experiment. Animals were kept at an ambient temperature of 25±1 °C and relative humidity of 50±5 % under a 12 h/12 h light/dark cycle. They were given standardized pellet food and water ad libitum.

### Experimental treatments and protocol

After 1 week of acclimatization, the mice were randomized on basis of body weight and allocated to group I-arsenic (As III), group II-arsenic and piperine co-administration (As +Pp), group III-piperine per se and group IV-control (*n*=4–5 per group). Meta sodium arsenite was dissolved in distilled water to make 50 ppm solution and mice in the As(III) and As +Pp groups were given access to this drinking water. Piperine (10 mg/kg) was administered orally in As +Pp and in the piperine per se group. Corn oil was used as a vehicle to administer piperine. The arsenic group received corn oil (0.1 ml/10 gm body weight) to nullify the effect of administered volume of various treatments. The control group was given access to distilled water.

### Collection of cecal content

After completion of the 120 days of the experiment, mice were sacrificed by cervical dislocation. Gut from the base of oesophagus to the rectum was isolated. Cecum content was rapidly isolated and total genomic DNA was isolated from a limited number of samples using a DNA stool mini kit (Qiagen, Hieldburg, Germany) and was kept at −80 °C until analyses. Approximately 8–10 µg purified genomic DNA with absorbance ratio 1.8–2.0 at the concentration of 500 ng µl^−1^ was used for V3-V4 region based 16S metagenomic sequencing (MiseQ-Illumina platform). Raw reads up to ~150 Mb per sample with an average read length of 2×250 bp were generated. PCR amplification was carried out using the KAPA HiFi HotStart DNA Polymerase (Kappa Biosystems). The 16S V3-V4 region was amplified using the forward primer (5′ CCTACGGGNGGCWGCAG 3′) and reverse primer (5′ GACTACHVGGGTATCTAATCC 3′). The thermal cycling conditions included an initial denaturation at 95 °C for 3 min, followed by 25 cycles of denaturation at 95 °C for 30 s, annealing at 55 °C for 30 s, and extension at 72 °C for 30 s. A final extension step at 72 °C for 5 min was performed.

The input DNA for library preparation was quantified using the Qubit dsDNA BR assay kit (Invitrogen). The quantification of 16S amplicon libraries was performed and PCR products were purified using Agencourt AMPure XP beads (Beckman Coulter) according to the manufacturer’s instructions. Library preparation was carried out using the Nextera XT index kit (Illumina). The quality and size distribution of the libraries were evaluated using the High Sensitivity DNA kit on the 2100 Bioanalyser system (Agilent). The purified and quantified amplicon libraries were sequenced using the MiSeq reagent kit V2 500 cycles on the MiSeq system (Illumina). The sequencing data were analysed utilizing bioinformatics tools like QIIME2, MG-RAST, STAMP and MicrobiomeAnalyst and taxonomically categorized [11]. Diversification, relative abundance and operational taxonomic unit based identification was determined.

### Results and discussion

**Fig. 1.**
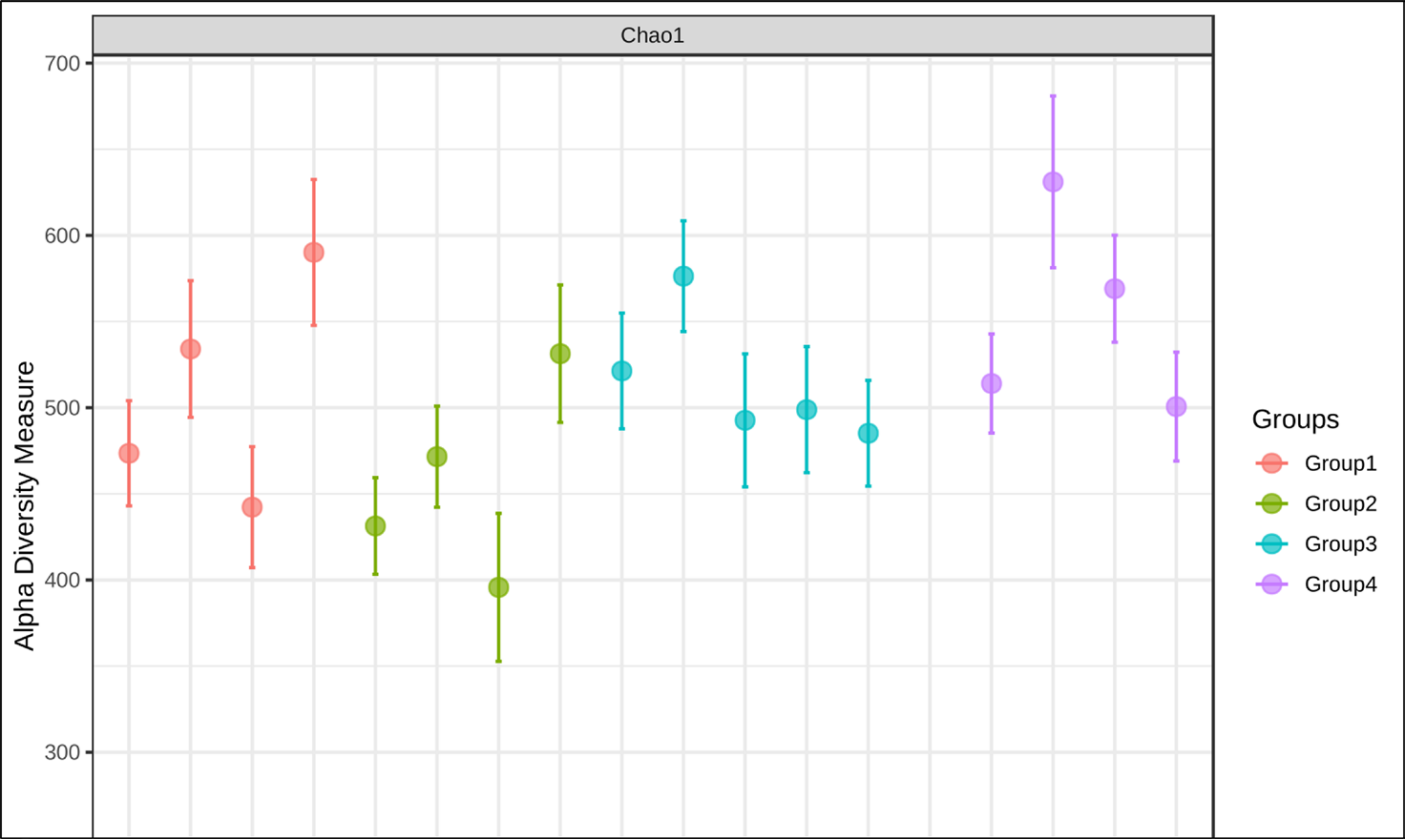
Alpha diversity index.

**Fig. 2.**
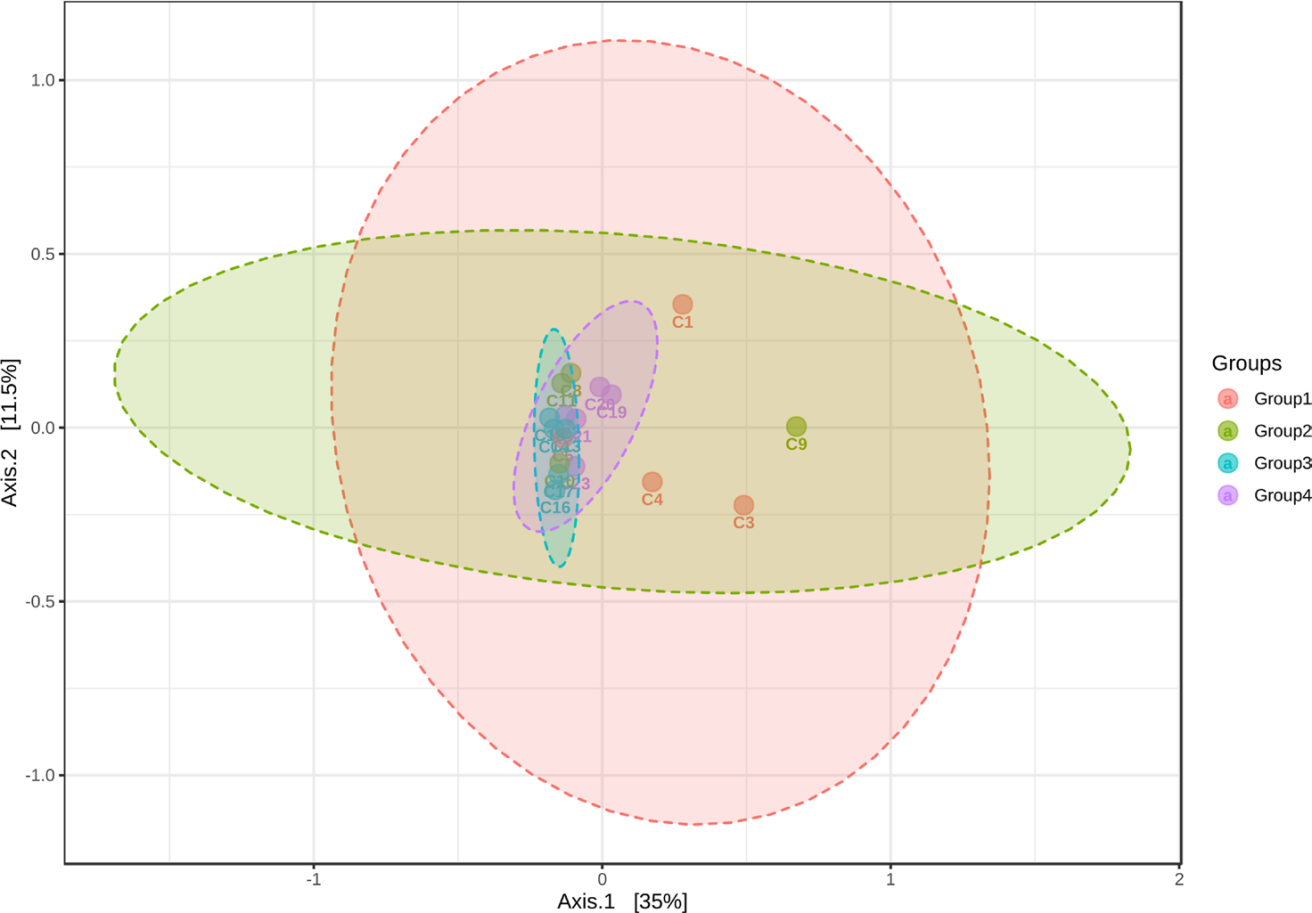
Beta diversity profiling.

The gut microbiome plays a vital part in heavy metal biotransformation, which can either enhance or mitigate the toxicity associated [12]. Dysbiosis of gut flora by metal exposure can adversely affect human health [13]. Changes in the bacterial community could seriously affect the host gut microbiota which has the potential to attribute to the pathogenesis of a spectrum of prevalent metabolic conditions such as non-alcoholic fatty liver disease, type two diabetes, obesity and cardio-metabolic diseases and neurological disorders [1, 14–16]. In our studies, we used cecal samples from four intervention groups arsenic exposure (As(III)), arsenic and piperine co-administration (As +Pp), piperine per se, and control group to examine the makeup and variety of intestinal bacteria. We found that the different intervention groups indicated an estimation of higher coverage of species or operational taxonomic units as shown in alpha diversity index-chao1 (Fig. 1). The beta diversity profiling using principal coordinate analysis-Bray Curtis index showed that As(III) group samples were found to be distinct from the other groups and have microbial compositions that set it apart from the other groups. Arsenic co-administered with piperine exhibit overlap to piperine per se group and in a close proximity to control group (Fig. 2).

**Fig. 3.**
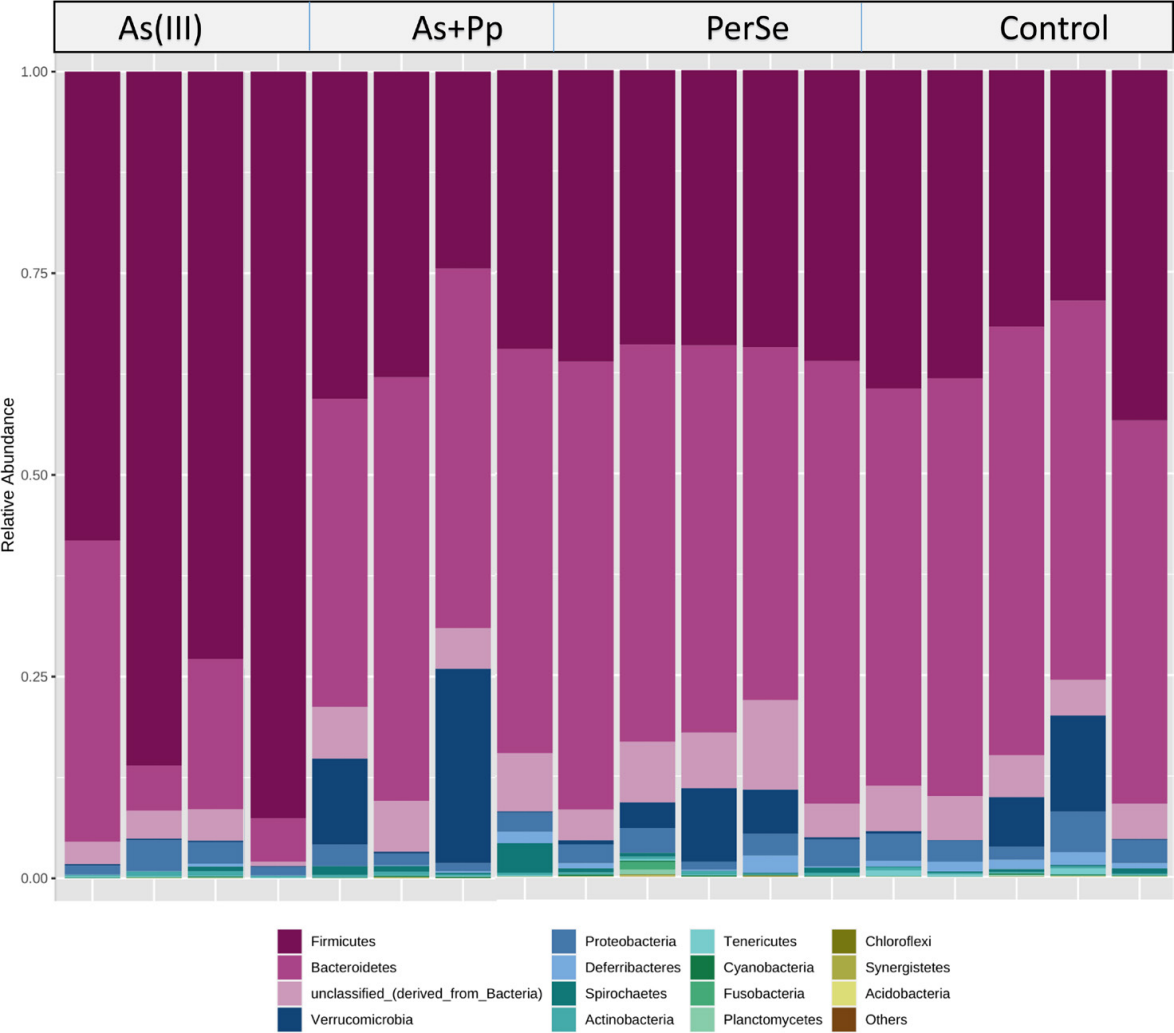
Relative abundance (percentage) of bacterial diversity at phylum level.

Further, variations in microbial abundance and composition across different taxonomic levels were observed. The human gut bacteria can be majorly assigned to four phyla being Firmicutes, Bacteroidetes, Actinobacteria, and Proteobacteria. In our studies, bacterial diversity at the phylum level showed higher relative abundance of Firmicutes and lower Bacteriodetes in As(III) group as compared to other intervention groups which is possibly due to toxicity or stress caused by heavy metal exposure. Conversely, As +Pp, per se and control group exhibited a relatively higher composition of Bacteroidetes. The former contribute to the release of energy from dietary fibre, which are a major source of propionate, whereas the latter is known to degrade carbohydrates and supply nutrients to the host and surrounding microbes that contribute to protection from pathogens and is associated with the host’s metabolism [17]. Proteobacteria and Verrucomicrobia were also observed in arsenic and piperine co-administered along with control group. However, Actinobacteria and Spirochaetes were present in relatively negligible proportions across each group (Fig. 3).

**Fig. 4.**
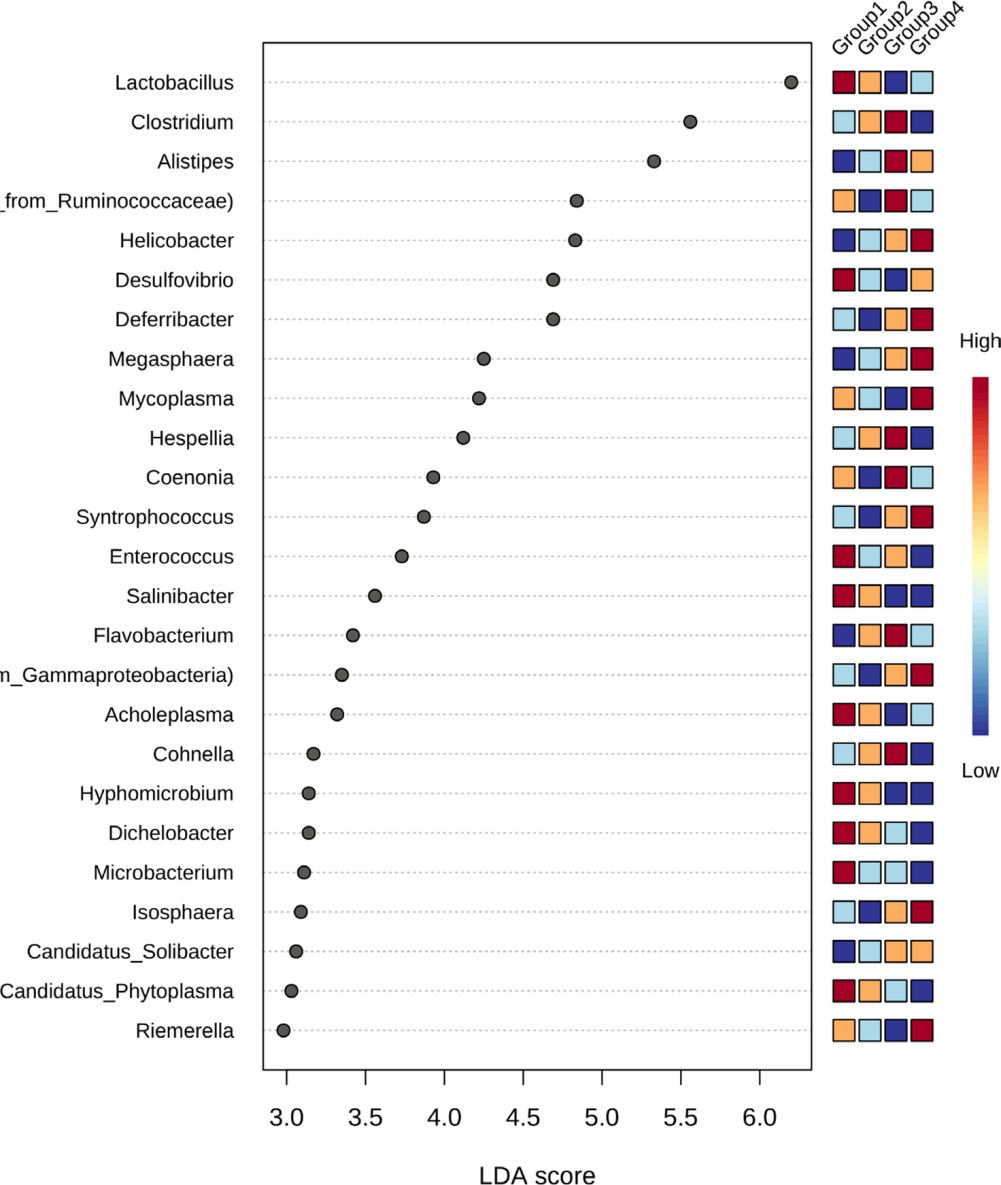
Lefse-Genus.

Next, we found that Lactobacillus, Desulfovibrio, Entercoccus, Salinibacter, Acholeplasma, Hyphomicrobium and Dichelobacter were predominantly present in As(III) group as compared to other groups. Lactobacillus is a notable *Firmicutes* genus, known as a natural inhabitant of the human gut with positive effects on human health and with inorganic arsenic adsorption capability leading to reduction of arsenic induced toxicity [18]. Functionally it also contributes to the metabolism of lipids, choline, and bile acid [19]. *Desulfovibrio* and *Enterococcus* are known as arsenic-metabolizing bacteria [20] which were predominantly seen in the metal exposure group. *Salinibacter* are known to code proteins that render arsenic resistance in high altitude hypersaline environments [21]. *Hyphomicrobium* are of environmental interest since many species can mineralize pollutants such as aromatic hydrocarbons, methyl chloride, and various alcohols [22]. Recently, a new genus *Arsenicitalea* of the family Hyphomicrobiaceae which is arsenic-resistant bacterium was isolated from the high-arsenic sediment [23]. *Clostridium*, a genus of the phylum Firmicutes, was found to be very less in metal exposure group as shown in linear discriminant analysis effect size (LDA) (Fig. 4). Clostridia as a group have been demonstrated to induce beneficial immune responses, in part via their ability to produce short-chain fatty acids that can attenuate gut inflammation [24].

## Conclusion

Overall, we observed the high operational taxonomical units in different interventions and control group. A decrease in Firmicutes phylum as a consequence of heavy metal induced toxicity or stress was observed. It further reduced the abundance of Bacteroidetes which are important for maintaining a healthy intestine and are involved in nutrient and energy absorption metabolism, immune system regulation, metabolic syndrome and gut-brain axis. Further, we observed the relatively higher enrichment of arsenic-metabolizing bacteria like Desulfovibrio, Salinibacter and Enterococcus in arsenic exposure group. Clostridium also showed a decreased presentation in this group. On the other hand, piperine co-administration brought the Bacteroidetes abundance normal to control group. These findings showed significant microbial diversity patterns across the experimental groups, highlighting the uniqueness of As(III) group and the similarity of As +Pp, piperine per se and control groups. However, deeper investigation is needed to further validate the eubiotic potential of piperine. Moreover, mice and humans have differences in anatomy, physiology, and immune systems. The controlled laboratory environment in which mice are typically housed may not accurately reflect the complexity of the human environment. Factors such as stress, environmental exposures, and the presence of specific pathogens can differ between lab mice and humans. Therefore, findings in mouse models may not always accurately represent human outcomes hence the studies further needs to be validated on human models.
